# Intensive pharmacist-led guideline-directed medical therapy for hospitalized heart failure patients: a single-center retrospective cohort study

**DOI:** 10.1186/s40780-026-00616-9

**Published:** 2026-08-03

**Authors:** Hikaru Katagiri, Komei Tanaka, Ryuji Kobayashi, Mizuki Tsuruta, Tetsuya Hasegawa, Yosuke Kawakami, Koji Muto, Hiroko Tanaka, Nobuhiro Sato, Keiichi Tsuchida, Kazuyoshi Takahashi

**Affiliations:** 1 Department of Pharmacy, Niigata City General Hospital, 463-7 Shumoku, Chuo-ku, Niigata, 950-1197 Japan; 2 Department of Cardiology, Niigata City General Hospital, 463-7 Shumoku, Chuo-ku, Niigata, Japan; 3 Department of Emergency and Critical Care Medicine, Niigata City General Hospital, 463-7 Shumoku, Chuo-ku, Niigata, Japan; 4 Department of Cardiology, Niigata Prefectural Shibata Hospital, 1-2-8 Honcho, Shibata, Japan

**Keywords:** Pharmacist, HFrEF, Heart failure, Guideline-directed medical therapy, Checklist

## Abstract

**Background:**

Guideline-directed medical therapy (GDMT) improves outcomes in heart failure with reduced ejection fraction (HFrEF). However, implementation remains suboptimal, and evidence for inpatient pharmacist-led interventions is limited. We evaluated the association between a pharmacist-led electronic medical record (EMR)-embedded checklist and GDMT use in hospitalized patients with acute heart failure.

**Methods:**

A single-center, retrospective, non-randomized study with a historical control group was conducted. Adult patients hospitalized with acute heart failure from October 2021 to March 2023 were enrolled. Patients admitted between October 2022 and March 2023 formed the pharmacist-led GDMT checklist intervention group, whereas those admitted between October 2021 and March 2022 served as historical controls. Primary outcomes were discharge implementation and new initiation rates of individual GDMT agents (renin–angiotensin system inhibitors, beta-blockers, mineralocorticoid receptor antagonists [MRA], sodium–glucose cotransporter-2 inhibitors [SGLT2i]) and quadruple therapy. Exploratory multivariable logistic regression was performed for quadruple therapy use at discharge in patients with HFrEF. Secondary outcomes included target-dose achievement, exploratory 90-day heart failure readmission, and all-cause mortality.

**Results:**

A total of 103 patients were included (49 controls, 54 intervention). Among patients with HFrEF (22 controls, 23 intervention), the intervention group had higher prescription rates of MRA (87.0% vs. 59.1%, *p* = 0.047) and SGLT2i (95.7% vs. 72.7%, *p* = 0.047). Quadruple therapy was achieved in 78.3% vs. 40.9% (*p* = 0.016). The proportion of patients who newly achieved quadruple therapy during hospitalization was also higher in the intervention group (83.3% vs. 35.0%, *p* = 0.038). In the exploratory multivariable model, the intervention remained associated with higher odds of quadruple therapy use at discharge. Target-dose achievement was similar between groups. In mildly reduced/preserved EF, discharge SGLT2i use tended to be higher in the intervention group (*p* = 0.062). Exploratory 90-day outcomes showed no clear between-group differences and were limited by incomplete follow-up and insufficient statistical power.

**Conclusions:**

The pharmacist-led EMR-embedded checklist intervention was associated with higher discharge implementation and in-hospital initiation of HFrEF-directed GDMT, particularly MRA, SGLT2i, and quadruple therapy. This simple checklist-based approach may support evidence-based pharmacotherapy optimization and warrants confirmation in larger multicenter studies with long-term follow-up.

**Supplementary Information:**

The online version contains supplementary material available at https://doi.org/10.1186/s40780-026-00616-9.

## Background

Heart failure is a public health challenge affecting approximately 26 million people worldwide [1]. In Japan, the number of patients with heart failure is expected to peak at 1.32 million by 2035, largely driven by an aging population [2]. The growing burden of heart failure not only strains healthcare systems but also leads to escalating healthcare expenditures, prompting concerns about an impending “heart failure pandemic” [3]. Consequently, minimizing hospitalizations and preventing recurrent decompensation are critical clinical priorities. The pharmacological management of heart failure with reduced ejection fraction (HFrEF) has advanced significantly in recent years. Current guideline-directed medical therapy (GDMT) includes four drug classes: renin–angiotensin system inhibitors (RASi), such as angiotensin-converting enzyme inhibitors (ACEi), angiotensin receptor blockers (ARB), or angiotensin receptor neprilysin inhibitors (ARNI); beta-blockers (BB); mineralocorticoid receptor antagonists (MRA); and sodium–glucose cotransporter-2 inhibitors (SGLT2i) [4]. The combination of these agents has been shown to improve survival outcomes. Despite strong recommendations from major guidelines [5, 6], GDMT implementation in real-world settings remains suboptimal, with the underuse of indicated therapies and low target-dose achievement [7].

To improve adherence, multidisciplinary approaches have been reported to be effective in optimizing heart failure care [8, 9]. Pharmacists play an essential role in this context by contributing to medication selection, dose titration, patient education, and monitoring of adverse effects. Pharmacists’ involvement can enhance GDMT adherence and reduce heart failure-related events [10, 11]. However, most evidence pertains to outpatient settings [12, 13], with limited data on inpatient pharmacist-led interventions, particularly in Japan.

This study investigated the association between a pharmacist-led GDMT intervention using a checklist and GDMT implementation in patients hospitalized with heart failure.

## Methods

### Study design and patient population

This single-center, retrospective, non-randomized study with a historical control group included adult patients (≥18 years) hospitalized with acute heart failure at the Niigata City General Hospital between October 2021 and March 2023. The pharmacist-led GDMT checklist was initiated in October 2022. Patients admitted between October 2022 and March 2023 comprised the intervention group, whereas those admitted between October 2021 and March 2022 served as historical controls without checklist use. Patients were included if they were admitted to the cardiology department, had a primary diagnosis of acute heart failure, and had participated in a heart failure rehabilitation program. Clinical data, including demographics, hospitalization details, laboratory results, echocardiographic parameters, comorbidities, and medication use, were extracted from the electronic medical record (EMR) system. Exclusion criteria included in-hospital death, transfer to another department, surgical intervention, an estimated glomerular filtration rate (eGFR) <20 mL/min/1.73 m^2^, or missing left ventricular ejection fraction (LVEF) data. The study was approved by the institutional review board (Approval No. 24-091).

### Heart failure classification and GDMT definitions

LVEF was measured at admission by transthoracic echocardiography using the 2D or Simpson’s method. HFrEF was defined as LVEF < 40% and GDMT comprised RASi (ACEi/ARB/ARNI), BB, MRA, and SGLT2i. Heart failure with mildly reduced ejection fraction (HFmrEF) and heart failure with preserved ejection fraction (HFpEF) (LVEF ≥ 40%) were grouped together. In this study, the terms “GDMT” and “quadruple therapy” were defined for HFrEF. For HFmrEF/HFpEF, we report medication use descriptively, with SGLT2i as the phenotype-appropriate, guideline-recommended therapy. The classification of heart failure based on the LVEF was in accordance with the clinical guidelines available at that time [14].

Patients were further classified into de novo and prior heart failure groups. De novo heart failure was defined as an index hospitalization for heart failure in patients without any prior history of heart failure. Prior heart failure was defined as hospitalization for worsening heart failure in patients with a documented history of heart failure before the index admission, regardless of previous hospitalizations.

### Pharmacist intervention

Pharmacists developed individualized GDMT checklists embedded in the EMR to promote adherence among multidisciplinary care teams (physicians, nurses, physical therapists, and dietitians). The checklist was initiated for patients who met all of the following criteria: (1) admission to the cardiology department for acute heart failure, (2) “heart failure” listed as the primary diagnosis at admission, and (3) enrollment in a heart failure rehabilitation program.

**Fig. 1:**
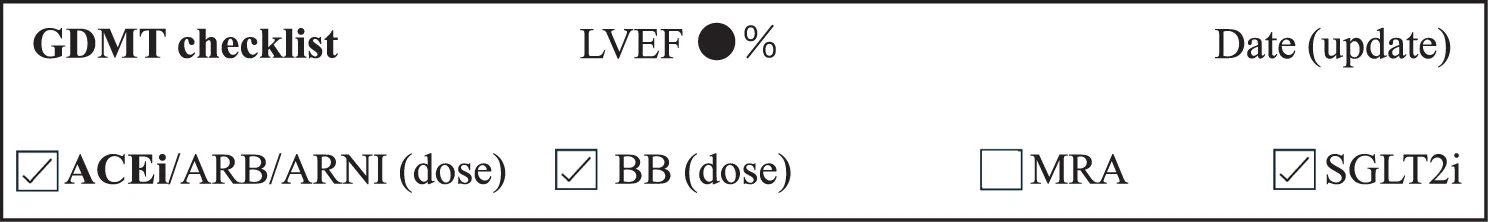
Example of a GDMT checklist (primarily for HFrEF). The checklist includes items such as LVEF, the checklist update date, and the GDMT implementation status. If ACEi, BB, and SGLT2i are prescribed, they are shown as illustrated in the figure. In addition, for RASi (ACEi/ARB/ARNI) and BB, the prescribed dosage should also be recorded. This checklist was primarily designed for patients with HFrEF, and partially applied to HFmrEF/HFpEF according to guideline recommendations. For this group, SGLT2i are highlighted as a phenotype-appropriate, guideline-recommended therapy. ACEi, angiotensin-converting enzyme inhibitor; ARB, angiotensin receptor blocker; ARNI, angiotensin receptor neprilysin inhibitor; BB, beta-blocker; GDMT, guideline-directed medical therapy; LVEF, left ventricular ejection fraction; MRA, mineralocorticoid receptor antagonist; RASi, renin–angiotensin system inhibitor; SGLT2i, sodium–glucose cotransporter-2 inhibitor

The checklist recorded the LVEF, checklist update date, GDMT implementation status, and drug dosing (only RASi and BB) (Fig. 1). Additional items, such as renal function and reasons for non-initiation, were added at the pharmacist’s discretion, as deemed clinically relevant. Pharmacists proposed the initiation of missing GDMT components and monitored adverse effects by updating checklists approximately two to three times a week.

We also evaluated process indicators of checklist implementation in the intervention group, including (1) the proportion of eligible hospitalizations in which a GDMT checklist was created in the EMR, (2) whether the checklist was entered at the intended time during hospitalization, and (3) the number and acceptance of direct GDMT-related prescription recommendations made by pharmacists.

### Study outcomes

The primary outcomes were the implementation rates at discharge of each GDMT agent and the rate of quadruple therapy (defined for HFrEF). To capture in-hospital implementation, we also assessed new initiation rates of each agent, defined as the proportion of patients who were not receiving the agent at admission but were newly started on that agent during hospitalization. Furthermore, to characterize barriers to GDMT implementation in patients with HFrEF, we descriptively summarized the reasons for the non-initiation of each class. We also summarized the reasons for the non-initiation of SGLT2i in patients with HFmrEF/HFpEF based on the information documented in the EMR.

Secondary pharmacological outcomes included the ARNI implementation rate, ARNI dosing, and the proportion of patients achieving ≥50% of the guideline-recommended target doses for ACEi/ARB and BB among patients with HFrEF. ARNI dosing was evaluated as the percentage of the guideline-recommended target dose at discharge. In addition, we descriptively summarized the distribution of % target dose and target-dose achievement for MRA and SGLT2i. The target doses for ACEi, ARB, BB, and MRA were based on the Japanese heart failure management guidelines available at the time of the study [14], whereas for ARNI and SGLT2i, target doses were defined according to international heart failure guidelines [5, 6]. Additional secondary clinical outcomes were 90-day heart failure readmission and 90-day all-cause mortality after hospital discharge, evaluated among patients with available 90-day follow-up data.

### Statistical analysis

Categorical variables were analyzed using Fisher’s exact test. Continuous variables were assessed for normality using the Shapiro–Wilk test. When both groups showed normal distributions, variance equality was tested using the F-test, followed by Student’s or Welch’s t-test, as appropriate. If normality was violated, the Mann–Whitney U test was applied. In addition, days to first readmission were summarized as median [interquartile range] and compared between groups using the Mann–Whitney U test. To assess the baseline balance between groups, we calculated standardized mean differences (SMDs) for each covariate. Absolute SMD values of ≥0.10 were interpreted as indicating potentially meaningful imbalance.

We performed an exploratory multivariable logistic regression analysis for the primary outcome of quadruple therapy use at discharge in patients with HFrEF. The dependent variable was receipt of quadruple therapy at discharge and the main independent variable was assignment to the intervention group (vs. control). Based on the clinical relevance and limited sample size, a history of ischemic heart disease and LVEF were included as covariates. Odds ratios (ORs) and 95% confidence intervals (CIs) were estimated using logistic regression with robust standard errors. Other baseline variables were evaluated in univariable logistic regression models only. To further explore potential effect modification, stratified analyses were performed for quadruple therapy use at discharge in patients with HFrEF according to age group (≥75 vs. <75 years), sex (female vs. male), and renal function (eGFR ≥ 45 vs. <45 mL/min/1.73 m^2^). The unadjusted ORs and 95% CIs for the association between the intervention and quadruple therapy were obtained from separate logistic regression models within each stratum.

*p* values for secondary outcomes, multivariable analyses, and subgroup/stratified analyses were considered descriptive and were not adjusted for multiplicity. All statistical analyses were performed using EZR version 1.68 [15], with *p* values < 0.05 considered statistically significant.

## Results

**Fig. 2:**
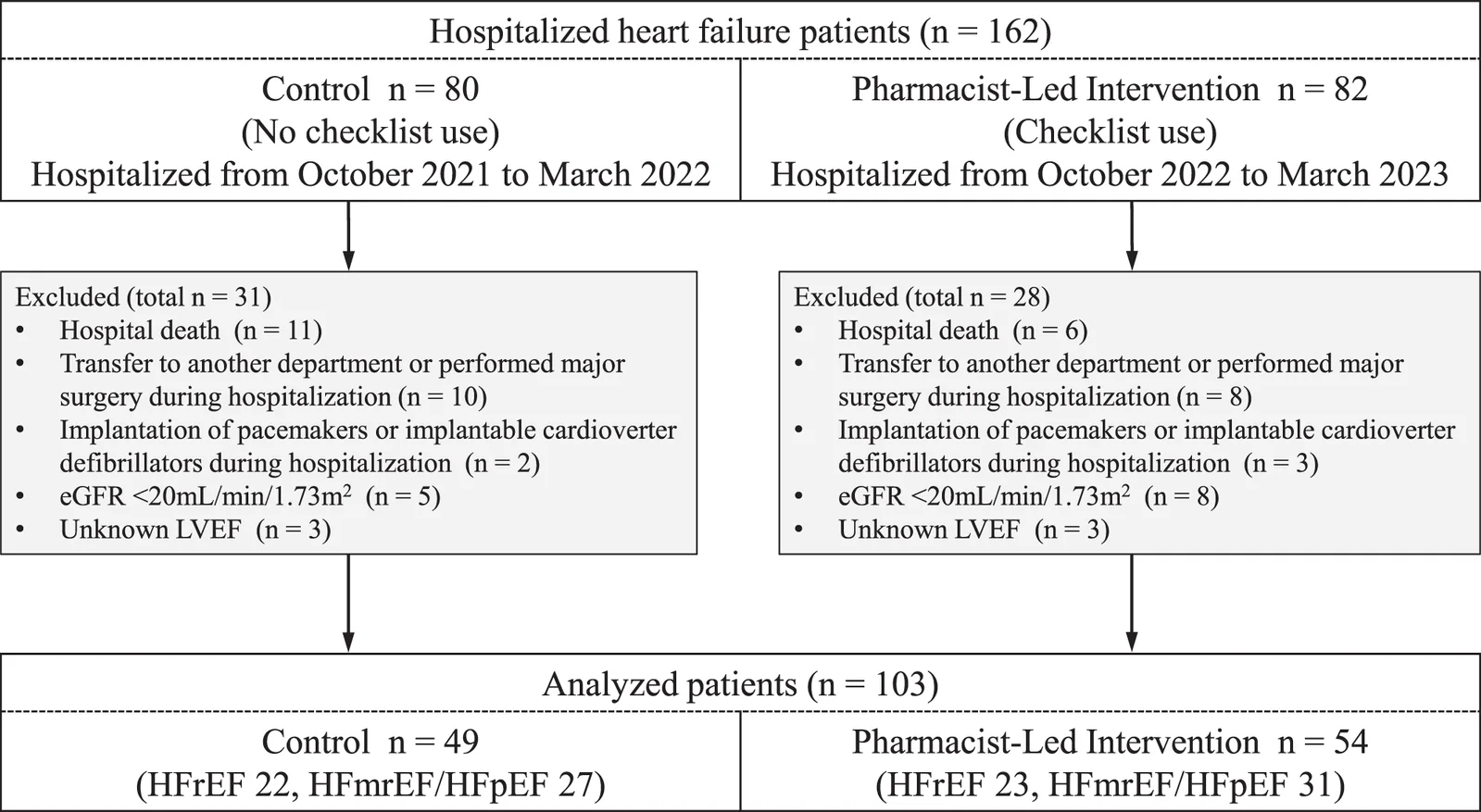
Patient selection and exclusion criteria. eGFR, estimated glomerular filtration rate; HFmrEF, heart failure with mildly reduced ejection fraction; HFpEF, heart failure with preserved ejection fraction; HFrEF, heart failure with reduced ejection fraction; LVEF, left ventricular ejection fraction

A total of 49 patients in the control group and 54 patients in the intervention group were included in the final analysis after excluding ineligible cases (Fig. 2).

Across analyses, discharge status was not interpreted in isolation; we also emphasize in-hospital initiation of GDMT agents.

In the intervention group, a GDMT checklist was created for 23/23 (100%) patients with HFrEF and 30/31 (96.8%) patients with HFmrEF/HFpEF, corresponding to 53/54 (98.1%) of eligible hospitalizations. All checklists were created at the intended time during the index hospitalization. In addition to the information provided through the checklist, pharmacists documented one direct GDMT-related prescription recommendation for patients with HFrEF (accepted in 1/1 case) and two recommendations for patients with HFmrEF/HFpEF (accepted in 1/2 cases). Details of these process indicators are presented in Supplementary Table S1.

### HFrEF

#### Baseline characteristics

The control and intervention groups included 22 and 23 patients, respectively. The patient characteristics are presented in Table 1. The groups were comparable in age and sex. The prevalence of ischemic heart disease was higher in the intervention group (43.5%) than in the control group (18.2%). Median LVEF was significantly higher in the intervention group (33% vs. 26%, *p* = 0.011), and median brain natriuretic peptide (BNP) level was lower (942.3 vs. 1714.7 pg/mL, *p* = 0.017). No significant differences were observed in eGFR or length of hospital stay. Baseline use of GDMT agents at admission (RASi, BB, MRA, and SGLT2i) is summarized in Table 2. Although baseline prescription rates differed numerically between periods, none of these differences were statistically significant.

**Table 1:** Patient characteristics

Characteristic	HFrEF (n = 45)				HFmrEF/HFpEF (n = 58)			
	Control (n = 22)	Pharmacist-Led Intervention (n = 23)	*p * value	SMD	Control (n = 27)	Pharmacist-Led Intervention (n = 31)	*p* value	SMD
Age (years)	78 (37–92)	76 (38–89)	0.900^a^	0.070	78 (51–95)	84 (54–100)	0.194^c^	0.345
Female (%)	7 (31.8)	7 (30.4)	1^b^	0.030	13 (48.1)	16 (51.6)	1^b^	0.069
BMI (kg/m^2^)	22.1 (13.1–25.5)	20.3 (13.1–42.5)	0.555^a^	0.121	23.1 (15.3–30.5)	22.1 (14.9–34.1)	0.877^c^	0.041
Na (mmol/L)	140 (132–144)	140 (131–145)	0.323^d^	0.297	141 (126–145)	139 (130–145)	0.076^a^	0.246
K (mmol/L)	4.0 (2.7–5.1)	4.0 (3.2–5.5)	0.285^c^	0.322	4.0 (3.0–6.2)	4.1 (2.6–5.6)	0.482^a^	0.104
eGFR (mL/min/1.73 m^2^)	45 (24–95)	47 (19–76)	0.973^a^	0.055	55 (20–90)	46 (23–80)	0.044^c^	0.542
BNP (pg/mL)	1714.7 (206.6–4250.0)	942.3 (97.3–3688.6)	0.017^a^	0.794	630.3 (45.3–2515.1)	503.1 (133.5–1204.7)	0.073^a^	0.616
sBP (mmHg)	150 (87–206)	141 (91–210)	0.799^c^	0.076	159.3	152.1	0.460^c^	0.196
dBP (mmHg)	87 (51–135)	80 (49–180)	0.947^c^	0.020	88.9	88.5	0.938^c^	0.021
HR (b.p.m.)	110 (67–140)	96 (50–154)	0.268^c^	0.335	100.7	102.4	0.831^c^	0.057
LVEF (%)	26 (12–38)	33 (17–39)	0.011^a^	0.754	46 (40–75)	51 (40–79)	0.095^a^	0.286
Hypertension (%)	15 (68.2)	17 (73.9)	0.749^b^	0.127	21 (77.8)	25 (80.6)	1^b^	0.071
Dyslipidemia (%)	7 (31.8)	5 (21.7)	0.514^b^	0.229	8 (29.6)	15 (48.4)	0.184^b^	0.392
Diabetes mellitus (%)	6 (27.3)	5 (21.7)	0.738^b^	0.129	9 (33.3)	14 (45.2)	0.426^b^	0.244
De novo heart failure (%)	11 (50.0)	14 (60.9)	0.554^b^	0.220	21 (77.8)	18 (58.1)	0.162^b^	0.432
Prior hospitalization for heart failure in 1 year (%)	5 (22.7)	3 (13)	0.459^b^	0.255	2 (7.4)	1 (3.2)	0.593^b^	0.187
Ischemic heart disease (%)	4 (18.2)	10 (43.5)	0.108^b^	0.570	6 (22.2)	5 (16.1)	0.739^b^	0.155
AF/AFL (%)	9 (40.9)	7 (30.4)	0.542^b^	0.220	12 (44.4)	17 (54.8)	0.599^b^	0.209
Hospital stay (days)	23 (13–57)	25 (12–50)	1^a^	0.108	18 (8–33)	22 (9–50)	0.011^a^	0.685

Data are presented as the number of cases (%) or median (min–max)

^a^ Mann–Whitney U test; ^b^ Fisher’s exact test; ^c^ Student’s t-test; ^d^ Welch’s t-test

*p* values for comparisons between the control and pharmacist-led intervention groups

SMD; absolute SMD ≥ 0.10 was considered indicative of meaningful imbalance

AF, atrial fibrillation; AFL, atrial flutter; BMI, body mass index; BNP, brain natriuretic peptide; dBP, diastolic blood pressure; eGFR, estimated glomerular filtration rate; HFmrEF, heart failure with mildly reduced ejection fraction; HFpEF, heart failure with preserved ejection fraction; HFrEF, heart failure with reduced ejection fraction; HR, heart rate; K, potassium; LVEF, left ventricular ejection fraction; Na, sodium; sBP, systolic blood pressure; SMD, standardized mean difference

**Table 2:** Prescription of heart failure medications at admission and discharge in the control and pharmacist-led intervention groups

Medication	HFrEF (n = 45)			HFmrEF/HFpEF (n = 58)		
	Control (n = 22)	Pharmacist-Led Intervention (n = 23)	*p *value	Control (n = 27)	Pharmacist-Led Intervention (n = 31)	*p *value
At admission						
RASi (%)	9 (40.9)	11 (47.8)	0.767	15 (55.6)	17 (54.8)	1
BB (%)	10 (45.5)	9 (39.1)	0.767	10 (37)	14 (45.2)	0.599
MRA (%)	4 (18.2)	8 (34.8)	0.314	3 (11.1)	3 (9.7)	1
SGLT2i (%)	4 (18.2)	6 (26.1)	0.722	1 (3.7)	6 (19.4)	0.108
Quadruple therapy	2 (9.1)	5 (21.7)	0.665	1 (3.7)	1 (3.2)	1
At discharge						
ARNI (%)	11 (50.0)	12 (52.2)	1	4 (14.8)	7 (22.6)	0.518
RASi (%)	20 (90.9)	22 (95.7)	0.608	24 (88.9)	28 (90.3)	1
BB (%)	19 (86.4)	22 (95.7)	0.346	23 (85.2)	22 (71.0)	0.225
MRA (%)	13 (59.1)	20 (87.0)	0.047	16 (59.3)	17 (54.8)	0.795
SGLT2i (%)	16 (72.7)	22 (95.7)	0.047	12 (44.4)	22 (71.0)	0.062
Quadruple therapy	9 (40.9)	18 (78.3)	0.016	7 (25.9)	10 (32.3)	0.773
Loop diuretics (%)	17 (77.3)	20 (87.0)	0.459	19 (70.4)	27 (87.1)	0.193
TLV (%)	5 (22.7)	6 (26.1)	1	4 (14.8)	7 (22.6)	0.518
Potassium binders (%)	2 (9.1)	4 (17.4)	0.665	2 (7.4)	2 (6.5)	1

Data are presented as the number of cases (%)

*p* values were calculated using Fisher’s exact test

Note: “Quadruple therapy” applies to HFrEF; for HFmrEF/HFpEF, the four-class combination (RASi, BB, MRA, and SGLT2i) is shown descriptively

ACEi, angiotensin-converting enzyme inhibitor; ARB, angiotensin receptor blocker; ARNI, angiotensin receptor neprilysin inhibitor; BB, beta-blocker; HFmrEF, heart failure with mildly reduced ejection fraction; HFpEF, heart failure with preserved ejection fraction; HFrEF, heart failure with reduced ejection fraction; MRA, mineralocorticoid receptor antagonist; RASi, renin–angiotensin system inhibitor (ACEi/ARB/ARNI); SGLT2i, sodium–glucose cotransporter-2 inhibitor; TLV, tolvaptan

#### Pharmacological treatment and GDMT implementation in patients with HFrEF

**Fig. 3:**
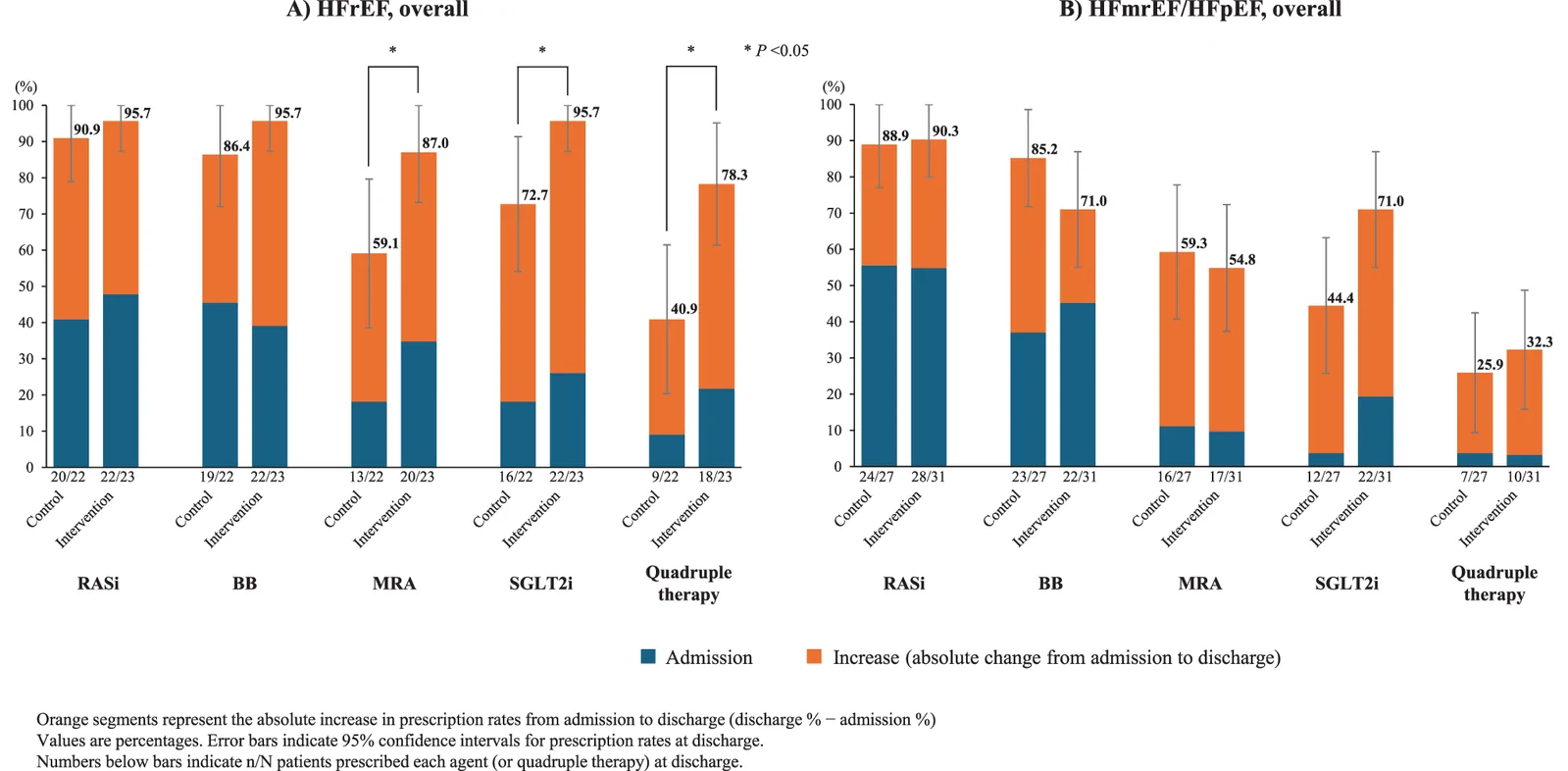
Prescription rates of heart failure medications at admission and discharge in the control group and the pharmacist-led checklist intervention group in the overall cohort. Panel a shows patients with HFrEF, and panel B shows patients with HFmrEF/HFpEF. Bars are stacked to show prescription rates at admission (bottom) and the absolute increase during hospitalization (top), with the total height representing prescription rates at discharge. Error bars indicate 95% confidence intervals (CIs) for prescription rates at discharge. Numbers below bars indicate n/N patients prescribed each agent (or quadruple therapy) at discharge.Note: “quadruple therapy” applies to HFrEF; for HFmrEF/HFpEF, the four-class combination (RASi, BB, MRA, and SGLT2i) is shown descriptively. ACEi, angiotensin-converting enzyme inhibitor; ARB, angiotensin receptor blocker; ARNI, angiotensin receptor neprilysin inhibitor; BB, beta-blocker; CI, confidence interval; HFmrEF, heart failure with mildly reduced ejection fraction; HFpEF, heart failure with preserved ejection fraction; HFrEF, heart failure with reduced ejection fraction; MRA, mineralocorticoid receptor antagonist; RASi, renin–angiotensin system inhibitor (ACEi/ARB/ARNI); SGLT2i, sodium–glucose cotransporter-2 inhibitor

Changes in GDMT prescription from admission to discharge in the overall HFrEF cohort are shown in Table 2 and illustrated in Fig. 3A as stacked bars indicating prescription rates at admission and the absolute increase during hospitalization. At discharge, the prescription rates for each GDMT component were consistently higher in the intervention group (RASi, 95.7%; BB, 95.7%; MRA, 87.0%; and SGLT2i, 95.7%) than in the control group (RASi, 90.9%; BB, 86.4%; MRA, 59.1%; and SGLT2i, 72.7%). MRA and SGLT2i showed statistically significant differences (*p* = 0.047). Quadruple therapy was achieved in 78.3% of the intervention group versus 40.9% in the control group (*p* = 0.016).

New initiation rates for each GDMT agent, defined as the proportion of patients not receiving the agent at admission who were newly started on that agent during hospitalization, were consistently higher in the intervention group than in the control group (Table 3). For example, the rate of new MRA initiation increased from 55.6% in the control group to 86.7% in the intervention group, and new initiation of SGLT2i increased from 66.7% to 100.0%. The proportion of patients newly achieving quadruple therapy was markedly higher with the intervention than with usual care (83.3% vs. 35.0%, *p* = 0.038), whereas between-group differences for individual agents did not reach statistical significance. Reasons for non-initiation of each GDMT component in patients with HFrEF are shown in Supplementary Table S2a.

**Table 3:** New initiation of GDMT during hospitalization in patients with HFrEF

GDMT	Control n/N (%)	Pharmacist-Led Intervention n/N (%)	*p* value
Overall			
RASi	11/13 (84.6)	11/12 (91.7)	1
BB	9/12 (75.0)	13/14 (92.9)	0.376
MRA	10/18 (55.6)	13/15 (86.7)	0.556
SGLT2i	12/18 (66.7)	17/17 (100.0)	0.221
Quadruple therapy	7/20 (35.0)	15/18 (83.3)	0.038
De novo HFrEF			
RASi	10/10 (100.0)	9/10 (90.0)	0.180
BB	7/8 (87.5)	12/13 (92.3)	0.350
MRA	7/11 (63.6)	10/12 (83.3)	1
SGLT2i	6/11 (54.5)	14/14 (100.0)	0.009
Quadruple therapy	4/11 (36.4)	10/14 (71.4)	0.116
Prior HFrEF			
RASi	1/3 (33.3)	2/2 (100.0)	0.566
BB	2/4 (50.0)	1/1 (100.0)	1
MRA	3/7 (42.9)	3/3 (100.0)	1
SGLT2i	6/7 (85.7)	3/3 (100.0)	0.406
Quadruple therapy	3/9 (33.3)	5/5 (100.0)	0.362

Data are presented as n/N (%). New initiation was defined as initiation of each GDMT agent during the index hospitalization among patients not receiving that agent at admission

*p* values were calculated using Fisher’s exact test

ACEi, angiotensin-converting enzyme inhibitor; ARB, angiotensin receptor blocker; ARNI, angiotensin receptor neprilysin inhibitor; BB, beta-blocker; GDMT, guideline-directed medical therapy; HFrEF, heart failure with reduced ejection fraction; MRA, mineralocorticoid receptor antagonist; RASi, renin–angiotensin system inhibitor (ACEi/ARB/ARNI); SGLT2i, sodium–glucose cotransporter-2 inhibitor

#### Exploratory multivariable and stratified analyses

In an exploratory multivariable logistic regression model adjusting for history of ischemic heart disease and LVEF, the pharmacist-led intervention remained associated with higher odds of receiving quadruple therapy at discharge (adjusted OR 5.13, 95% CI 1.10–24.00, *p* = 0.038), consistent with the univariate estimate (OR 5.20, 95% CI 1.41–19.20; Supplementary Table S3, logistic regression for quadruple therapy). We also performed stratified analyses based on age, sex, and renal function because the limited sample size did not allow simultaneous adjustment for additional clinically important variables. In these exploratory subgroup analyses, the odds of receiving quadruple therapy with the intervention were particularly high among patients aged ≥ 75 years (OR 17.50, 95% CI 2.67–115.00), female patients (OR 15.00, 95% CI 1.03–218.00), and those with eGFR ≥ 45 mL/min/1.73 m^2^ (OR 13.30, 95% CI 1.78–100.00), whereas the associations were weaker and not statistically significant in younger patients and in those with impaired renal function (Supplementary Table S4, stratified analyses by age, sex, and renal function).

#### De Novo HFrEF and HFrEF with prior heart failure

For de novo HFrEF, admission-to-discharge changes in GDMT prescription rates are summarized in Supplementary Table S5 (admission-to-discharge GDMT changes in de novo HFrEF) and illustrated in Supplementary Fig. S1A (GDMT changes in de novo HFrEF). Prescription rates increased in both groups, with a greater increase in the intervention group; however, SGLT2i use at discharge was higher in the intervention group (*p* = 0.009). New in-hospital initiation rates are summarized in Table 3. SGLT2i initiation was more frequent with the intervention (*p* = 0.009) and quadruple therapy initiation tended to be higher, although this difference was not statistically significant.

Regarding HFrEF with prior heart failure, admission-to-discharge changes are summarized in Supplementary Table S6 (admission-to-discharge GDMT changes in HFrEF with prior heart failure) and illustrated in Supplementary Fig. S2A (GDMT changes in HFrEF with prior heart failure). Baseline medication use at admission was generally higher than that in de novo HFrEF, reflecting ongoing pre-admission treatment. In-hospital initiation outcomes (Table 3) were better in the intervention group; however, this difference was not statistically significant, likely due to the small denominators.

#### Secondary pharmacological outcomes

Secondary pharmacological outcomes for HFrEF are summarized in Tables 2 and 4. At discharge, ARNI use was similar between the groups (52.2% vs. 50.0%; Table 2). Similarly, the distribution of % target dose and the proportion achieving ≥ 50% of the target dose were comparable between groups for ARNI, ACEi/ARB, BB, MRA, and SGLT2i (Table 4). For example, median % target dose for ACEi/ARB tended to be higher in the intervention group (37.5% vs. 25.0%), and a larger proportion of patients achieved ≥ 50% of the target dose (50.0% vs. 22.2%), although these differences did not reach statistical significance. For BB, median % target dose was 25.0% in both groups, and fewer than 40% of patients achieved ≥ 50% of the target dose in either group (36.8% in the control group vs. 27.3% in the intervention group). For MRA, approximately two-thirds of patients in both groups achieved ≥ 50% of the target dose (65.0% in the intervention group vs. 69.2% in the control group), and almost all patients prescribed SGLT2i in either group received the full guideline dose. Overall, the intervention substantially increased the use of each GDMT class at discharge, whereas up-titration to higher doses remained modest for RASi and BB.

**Table 4:** Distribution of % target dose and achievement of ≥50% target dose for each GDMT class at discharge in patients with HFrEF

Drug class	Group	n prescribed	% of target dose, median [IQR]	<25% n (%)	25–49% n (%)	50–74% n (%)	≥75% n (%)	≥50% of target dose, n (%)	*p* value
ARNI	Control	11	25.0 [25.0–50.0]	0 (0)	7 (63.6)	3 (27.3)	1 (9.1)	4 (36.4)	1
	Pharmacist-Led Intervention	12	25.0 [25.0–50.0]	0 (0)	7 (58.3)	3 (25.0)	2 (16.7)	5 (41.7)	
ACEi/ARB	Control	9	25 [25.0–33.0]	2 (22.2)	5 (55.6)	2 (22.2)	0 (0)	2 (22.2)	0.350
	Pharmacist-Led Intervention	10	37.5 [25.0–50.0]	0 (0)	5 (50.0)	5 (50.0)	0 (0)	5 (50.0)	
BB	Control	19	25 [15.6–62.5]	6 (31.6)	5 (26.3)	3 (15.8)	5 (26.3)	7 (36.8)	0.737
	Pharmacist-Led Intervention	22	25 [25.0–46.9]	3 (13.6)	13 (59.1)	5 (22.7)	1 (4.5)	6 (27.3)	
MRA	Control	13	50 [25.0–50.0]	0 (0)	4 (30.8)	7 (53.8)	2 (15.4)	9 (69.2)	1
	Pharmacist-Led Intervention	20	50 [25.0–50.0]	2 (10.0)	5 (25.0)	12 (60.0)	1 (5.0)	13 (65.0)	
SGLT2i	Control	16	100 [100.0–100.0]	0 (0)	0 (0)	0 (0)	16 (100.0)	16 (100.0)	–
	Pharmacist-Led Intervention	22	100 [100.0–100.0]	0 (0)	0 (0)	0 (0)	22 (100.0)	22 (100.0)	

Data are presented as median (IQR) or n (%)

*p* values are for comparisons of the proportion achieving ≥ 50% of target dose between groups (Fisher’s exact test)

ACEi, angiotensin-converting enzyme inhibitor; ARB, angiotensin receptor blocker; ARNI, angiotensin receptor neprilysin inhibitor; BB, beta-blocker; GDMT, guideline-directed medical therapy; HFrEF, heart failure with reduced ejection fraction; IQR, interquartile range; MRA, mineralocorticoid receptor antagonist; SGLT2i, sodium–glucose cotransporter-2 inhibitor

#### Clinical outcomes

The 90-day post-discharge clinical outcomes for patients with HFrEF are summarized in Table 5. Among the 45 patients with HFrEF, 90-day follow-up data for heart failure readmission and mortality were available for 14 patients in the control group and 17 in the intervention group. Within this subset, 90-day heart failure readmission occurred in 3/14 (21.4%) patients in the control group and 4/17 (23.5%) in the intervention group, and no deaths were observed within 90 days after discharge in either group. Readmissions were related to worsening heart failure or issues with adherence to or continuation of GDMT, although some cases had unknown causes. Baseline characteristics according to the availability of 90-day follow-up data are shown in Supplementary Table S7. Among patients with HFrEF, those with available follow-up had slightly higher serum potassium levels (median 4.1 vs. 3.7 mmol/L, *p* = 0.009), and LVEF was lower in the available follow-up group than in those without follow-up (median 30.0% vs. 34.5%, *p* = 0.031), whereas other baseline characteristics were broadly similar between the groups.

**Table 5:** Exploratory 90-day post-discharge outcomes: heart failure readmission and all-cause mortality

Outcome	HFrEF (n = 31)			HFmrEF/HFpEF (n = 28)		
	Control (n = 14)	Pharmacist-Led Intervention (n = 17)	*p *value	Control (n = 16)	Pharmacist-Led Intervention (n = 12)	*p *value
90-day heart failure readmission, n/N (%)	3/14 (21.4)	4/17 (23.5)	1^a^	2/16 (12.5)	1/12 (8.3)	1^a^
Days to first readmission, median [IQR]	11 [8–40]	29 [19–39]	0.629^b^	44 [32–55]	12 [12–12]	0.667^b^
90-day all-cause mortality, n/N (%)	0/14 (0)	0/17 (0)	–^c^	0/16 (0)	0/12 (0)	–^c^

Patient numbers represent those with available 90-day follow-up data after excluding patients who were transferred to another hospital and those hospitalized for other reasons within 90 days after discharge

^a^ *p* values for readmission rates were calculated using Fisher’s exact test

^b^ Days to first readmission were summarized as median [IQR] among patients with 90-day heart failure readmission and compared between groups using the Mann–Whitney *U* test

^c^ *p* values for 90-day mortality were not calculated because no deaths occurred in either group

HFmrEF, heart failure with mildly reduced ejection fraction; HFpEF, heart failure with preserved ejection fraction; HFrEF, heart failure with reduced ejection fraction; IQR, interquartile range

### HFmrEF/HFpEF

#### Baseline characteristics

The control and intervention groups included 27 and 31 patients, respectively. The patient characteristics are presented in Table 1. No significant differences in age or sex were observed between control and intervention groups. Ischemic heart disease was present in 22.2% and 16.1% of the patients, respectively. The median LVEF was 46 and 51% in the control and intervention groups, respectively. BNP levels were similar between the groups. The eGFR was significantly lower in the intervention group (46 vs. 55 mL/min/1.73 m^2^, *p* = 0.044), and the median hospital stay was longer (22 vs. 18 days, *p* = 0.011). Baseline prescription rates of RASi, BB, MRA, and SGLT2i at admission did not differ significantly between the groups (Table 2).

#### Pharmacological treatment and SGLT2i use in patients with HFmrEf/hfpef

Changes in medication use from admission to discharge in patients with HFmrEF/HFpEF are summarized in Table 2 and illustrated in Fig. 3B as stacked bars showing prescription rates at admission and absolute increases during hospitalization. At discharge, the prescription rate of SGLT2i was higher in the intervention group (71.0%) than in the control group (44.4%), although the difference was not statistically significant (*p* = 0.062). Discharge prescription rates for RASi, BB, and MRA were broadly comparable between the two groups. The four-class combination (RASi, BB, MRA, and SGLT2i) was prescribed to 32.3% of patients in the intervention group and 25.9% in the control group, with no significant between-group differences.

New initiation rates for each medication class were generally similar between groups (Table 6). New initiation of SGLT2i tended to be more frequent in the intervention group than in the control group (64.0% vs. 42.3%); however, the difference was not statistically significant. Reasons for non-initiation of SGLT2i among patients with HFmrEF/HFpEF are shown in Supplementary Table S2b.

**Table 6:** New initiation of medication classes during hospitalization in patients with HFmrEF/HFpEF

Medication	Control n/N (%)	Pharmacist-Led Intervention n/N (%)	*p* value
Overall			
RASi	11/12 (91.7)	13/14 (92.9)	1
BB	13/17 (76.5)	10/17 (58.8)	0.285
MRA	13/24 (54.2)	15/28 (53.6)	1
SGLT2i	11/26 (42.3)	16/25 (64.0)	0.441
Quadruple therapy	7/26 (26.9)	9/30 (30.0)	1
De novo HFmrEF/HFpEF			
RASi	10/11 (90.9)	10/10 (100.0)	0.751
BB	13/16 (81.3)	9/12 (75.0)	0.528
MRA		10/18 (55.6)	1
SGLT2i	9/21 (42.9)	12/16 (75.0)	0.201
Quadruple therapy	5/21 (23.8)	5/21 (23.8)	1
Prior HFmrEF/HFpEF			
RASi	1/1 (100.0)	3/4 (75.0)	1
BB	0/1 (0.0)	1/5 (20.0)	1
MRA	2/4 (50.0)	5/10 (50.0)	1
SGLT2i	2/5 (40.0)	4/9 (44.4)	1
Quadruple therapy	2/6 (33.3)	4/12 (33.3)	1

Data are presented as n/N (%). New initiation was defined as initiation of each heart failure medication class during the index hospitalization among patients not receiving that medication class at admission

*p* values were calculated using Fisher’s exact test

Note: “Quadruple therapy” applies to HFrEF; for HFmrEF/HFpEF, the four-class combination (RASi, BB, MRA, and SGLT2i) is shown descriptively

ACEi, angiotensin-converting enzyme inhibitor; ARB, angiotensin receptor blocker; ARNI, angiotensin receptor neprilysin inhibitor; BB, beta-blocker; HFmrEF, heart failure with mildly reduced ejection fraction; HFpEF, heart failure with preserved ejection fraction; HFrEF, heart failure with reduced ejection fraction; MRA, mineralocorticoid receptor antagonist; RASi, renin–angiotensin system inhibitor (ACEi/ARB/ARNI); SGLT2i, sodium–glucose cotransporter-2 inhibitor

#### De Novo HFmrEF/HFpEF and HFmrEF/HFpEF with prior heart failure

In de novo HFmrEF/HFpEF, admission-to-discharge changes in heart failure medication prescription rates are summarized in Supplementary Table S5 (admission-to-discharge medication changes in de novo HFmrEF/HFpEF) and illustrated in Supplementary Fig. S1B (medication changes in de novo HFmrEF/HFpEF). Prescription rates increased in both groups, with a greater increase in the intervention group; discharge SGLT2i use was also higher in the intervention group (*p* = 0.049). New in-hospital initiation rates are summarized in Table 6. SGLT2i initiation rate was higher in the intervention group, with no clear between-group differences.

In HFmrEF/HFpEF with prior heart failure, admission-to-discharge changes are summarized in Supplementary Table S6 (admission-to-discharge medication changes in HFmrEF/HFpEF with prior heart failure) and illustrated in Supplementary Fig. S2B (medication changes in HFmrEF/HFpEF with prior heart failure). Baseline medication use at admission was generally higher than that in patients with de novo HFmrEF/HFpEF, reflecting ongoing pre-admission treatment. In-hospital initiation outcomes (Table 6) showed no clear between-group differences.

#### Clinical outcomes

The 90-day post-discharge clinical outcomes for patients with HFmrEF/HFpEF are summarized in Table 5. Among 58 patients with HFmrEF/HFpEF, 90-day follow-up data for heart failure readmission and mortality were available for 16 patients in the control group and 12 in the intervention group. Within this subset, 90-day heart failure readmissions occurred in 2/16 (12.5%) patients in the control group and 1/12 (8.3%) in the intervention group; no deaths were observed within 90 days after discharge in either group. In all patients who were readmitted, the cause of readmission was not clearly documented in the medical records. Baseline characteristics according to the availability of 90-day follow-up data for patients with HFmrEF/HFpEF are shown in Supplementary Table S7, and were generally comparable between those with and without follow-up.

## Discussion

This study evaluated the association between the pharmacist-led simple checklist–based intervention and GDMT implementation in patients with HFrEF. Compared with the control group, the intervention group had higher discharge prescription rates of MRA, SGLT2i, and quadruple therapy, along with a higher proportion of patients who newly achieved quadruple therapy during hospitalization. In addition, an exploratory multivariable logistic regression model adjusted for a history of ischemic heart disease and LVEF showed that the pharmacist-led intervention remained associated with higher odds of receiving quadruple therapy at discharge. Stratified analyses suggested larger associations in older patients, women, and those with preserved renal function.

Although previous reports have indicated that the use of checklists is associated with GDMT optimization and lower readmission rates [16], pharmacist-led initiatives remain limited. In this study, we implemented a checklist placed on the top page of each patient’s EMR to enhance multidisciplinary communication. This design was intended to enhance visibility during chart reviews and to promote collaborative care. We evaluated process indicators of checklist implementation; however, usage metrics such as how often the checklist was actually viewed or “completed” by physicians could not be assessed because our EMR system does not provide reliable audit-log data. Nevertheless, the checklist was placed on the top page of each patient’s EMR, and the cardiologists reported that it was readily visible and could be checked as part of their routine workflow. Based on these observations, the checklists were considered consistently available for review on the top page of the EMR, although actual viewing or use could not be objectively quantified. In practice, the checklist was created for almost all eligible hospitalizations, generally at the intended time, and pharmacists occasionally documented direct prescription recommendations, indicating that the intervention was feasible for incorporation into the routine clinical workflow. Potential drawbacks include reduced readability owing to information overload and inconsistent updates in the absence of a pharmacist. However, these issues did not appear to substantially hinder their implementation in routine practice.

In the HFrEF group, the intervention group showed significantly higher rates of quadruple GDMT, with MRA prescriptions increasing from 59.1% to 87.0%, and SGLT2i use increasing from 72.7% to 95.7%. Although MRA may be discontinued due to concerns over renal impairment and hyperkalemia [17], accumulating evidence supports its safety and benefits even in patients with renal dysfunction [18, 19]. The checklist may have supported the recognition of missing GDMT components and pharmacist-led adverse event monitoring was incorporated into the intervention process. Our implementation rates were notably high compared with those of large Japanese registry data, such as the Kyoto Congestive Heart Failure and Heart Institute of Japan Heart Failure II studies [20, 21], consistent with an association between intervention and higher implementation.

SGLT2i are recommended across the ejection fraction spectrum in contemporary heart failure guidance [22]. In this study, SGLT2i use increased markedly among patients with de novo HFrEF, with the new initiation rate reaching 100% in the intervention group. Among patients with HFmrEF/HFpEF, the overall SGLT2i use also increased from 44.4% to 71.0%, whereas in the de novo HFmrEF/HFpEF subgroup, the discharge prescription rate was higher in the intervention group than in the control group, although the new initiation rates did not differ significantly because of the small sample size. Taken together, these findings suggest that the intervention may be associated with broader SGLT2i use across the ejection fraction spectrum, particularly when baseline implementation is low.

However, our intervention was initiated after the publication of pivotal clinical trials supporting the use of SGLT2i [23–26], which must be considered when interpreting these results. Recent studies have reported a temporal upward trend in SGLT2i prescription among patients with acute heart failure [27]. The observed increase in quadruple GDMT implementation may partly reflect the accumulation of new clinical evidence and updates to practice guidelines that have heightened physicians’ awareness of GDMT optimization. Nevertheless, our findings also indicate that the intervention was associated with a higher prescription rate of MRA, which had already been recommended prior to the SGLT2i era. Although the between-group differences in MRA new initiation did not reach statistical significance, new initiation rates in the intervention group were consistently higher than in the control group in both de novo and prior heart failure subgroups, and we consider that the increase in MRA prescriptions may be one factor related to the observed increase in quadruple therapy use. Thus, despite the potential confounding effects of evolving clinical practice patterns over time, our findings suggest that the intervention was associated with greater GDMT implementation.

Regarding RASi and BB, prior evidence indicated a dose-dependent improvement in HFrEF outcomes [28]. Current guidelines recommend the early and aggressive initiation of ARNIs where appropriate [6]. However, this study did not observe a significant increase in ARNI use or in the doses of RASi or BB, and the distributions of the prescribed doses were similar between groups. These findings suggest that the checklist-based intervention was mainly associated with higher implementation of GDMT at discharge, whereas its association with dose optimization was limited. One possible reason why the ARNI implementation rate remained at approximately 50% is that the Japanese guidelines recommend switching from ACEi/ARB to ARNI only in symptomatic patients already receiving triple therapy (ACEi/ARB, BB, and MRA) [14]. This may possibly explain the limited initiation of ARNI during hospitalization.

The STRONG-HF trial demonstrated that rapid GDMT up-titration reduced both readmission and mortality rates [29]. In a Japanese study examining GDMT scores, higher scores were associated with lower mortality and readmission [30]. In the present study, the initiation of ARNI and up-titration of ACEi/ARB and BB were identified as challenges. Addressing these aspects may be important for GDMT score optimization and for improving clinical outcomes subsequently, although this hypothesis requires confirmation in future studies. Despite the longer average hospital stay in Japan than in other countries [31], which may provide opportunities for medication up-titration, the achievement of target GDMT doses before discharge remains suboptimal. Future strategies should consider approaches to facilitate safe and effective dose escalation during hospitalization.

We did not observe clear differences in 90-day heart failure readmission or mortality between the control and intervention groups despite higher GDMT implementation in the intervention group. In both the HFrEF and HFmrEF/HFpEF cohorts, the proportions of patients with 90-day heart failure readmission were similar and no deaths occurred within 90 days after discharge. These findings should be interpreted with caution, because the analyses were exploratory, based on a limited number of events, and restricted to patients with available 90-day follow-up data. In addition, our intervention focused solely on in-hospital optimization of GDMT and did not include a structured post-discharge management program, which may partly explain why no clear differences in short-term clinical outcomes were observed despite a higher prescribing rate at discharge. Taken together, these findings indicate that although our intervention was associated with higher GDMT implementation at discharge, the present study was not designed or powered to demonstrate definitive improvements in short-term post-discharge clinical or surrogate outcomes. Therefore, the 90-day heart failure readmission and mortality analyses should be interpreted as exploratory. Given the multiple secondary and subgroup analyses, *p* values should be interpreted descriptively, and these findings are hypothesis-generating.

### Limitations

Despite its contributions, this study has several limitations. First, as a single-center, retrospective, non-randomized study with a historical control group and a limited sample size, its extrapolation is restricted. Additionally, residual confounding due to unmeasured variables and temporal changes in clinical practice (including evolving evidence and guideline updates regarding GDMT and SGLT2i) cannot be excluded. In particular, the exploratory multivariable and stratified analyses of quadruple therapy in HFrEF were based on small numbers of patients and events and required parsimonious adjustment. Thus, findings from these secondary, exploratory multivariable, and stratified analyses, including their *p* values, should be interpreted as descriptive and hypothesis-generating rather than definitive. Second, the intervention was limited to the inpatient period and did not include a structured post-discharge management program. Moreover, 90-day post-discharge outcomes were available only for a subset of patients, and the number of events was small; therefore, the analyses of 90-day heart failure readmission and mortality were underpowered and should be interpreted as exploratory. In addition, post-discharge BNP, LVEF, and quality of life data were not systematically collected, precluding a robust evaluation of surrogate outcomes. Third, the frequency of checklist updates and content of therapeutic proposals for uninitiated agents were determined by individual pharmacists, potentially introducing variability into the fidelity of the intervention. Although checklist implementation and timing were nearly universal, our EMR system did not allow us to quantify usage metrics such as how often the checklist was merely viewed versus actively used, and the small number of explicitly documented pharmacist recommendations limited the depth of process evaluation. Fourth, because the final prescription decisions were made by attending physicians, it was not possible to eliminate selection bias, including potential differences in prescribing behavior between the historical control and intervention periods. Fifth, the impact of the checklist on patients with HFmrEF/HFpEF—particularly those with HFpEF—was constrained because there are fewer guideline-supported pharmacologic options than for HFrEF, and SGLT2i is recommended for this population. Therefore, additional evidence-based therapies for HFpEF are warranted.

## Conclusions

This study suggests that the pharmacist-led EMR-embedded checklist intervention was associated with a higher discharge implementation of HFrEF GDMT, particularly MRA, SGLT2i, and quadruple therapy, and a higher proportion of patients who newly achieved quadruple therapy during hospitalization. The exploratory 90-day clinical outcomes showed no clear between-group differences; however, these findings should be interpreted with caution because of the incomplete follow-up and limited statistical power. This simple and feasible approach may be a practical strategy to support evidence-based pharmacotherapy and warrants confirmation in larger, preferably multicenter, studies with long-term follow-up.

## Electronic supplementary material

Below is the link to the electronic supplementary material.

Supplementary Material 1

Supplementary Material 2

Supplementary Material 3

Supplementary Material 4

## Data Availability

The datasets generated and/or analyzed in the current study are available from the corresponding author upon reasonable request.

## Abbreviations

ACEi: Angiotensin-converting enzyme inhibitors
ARB: Angiotensin receptor blockers
ARNI: Angiotensin receptor neprilysin inhibitors
BB: Beta-blockers
BNP: Brain natriuretic peptide
eGFR: Estimated glomerular filtration rate
EMR: Electronic medical record
GDMT: Guideline-directed medical therapy
HFmrEF: Heart failure with mildly reduced ejection fraction
HFpEF: Heart failure with preserved ejection fraction
HFrEF: Heart failure with reduced ejection fraction
LVEF: Left ventricular ejection fraction
MRA: Mineralocorticoid receptor antagonists
RASi: Renin–angiotensin system inhibitors
SGLT2i: Sodium–glucose cotransporter-2 inhibitors
