# Supplementary material for: Intensive pharmacist-led guideline-directed medical therapy for hospitalized heart failure patients: a single-center retrospective cohort study
Source: J Pharm Health Care Sci. 2026 Aug 3;12:93. doi: 10.1186/s40780-026-00616-9 (PMC13726016; doi:10.1186/s40780-026-00616-9)
Supplement: Supplementary file 1 [file 40780_2026_616_MOESM1_ESM.pdf]

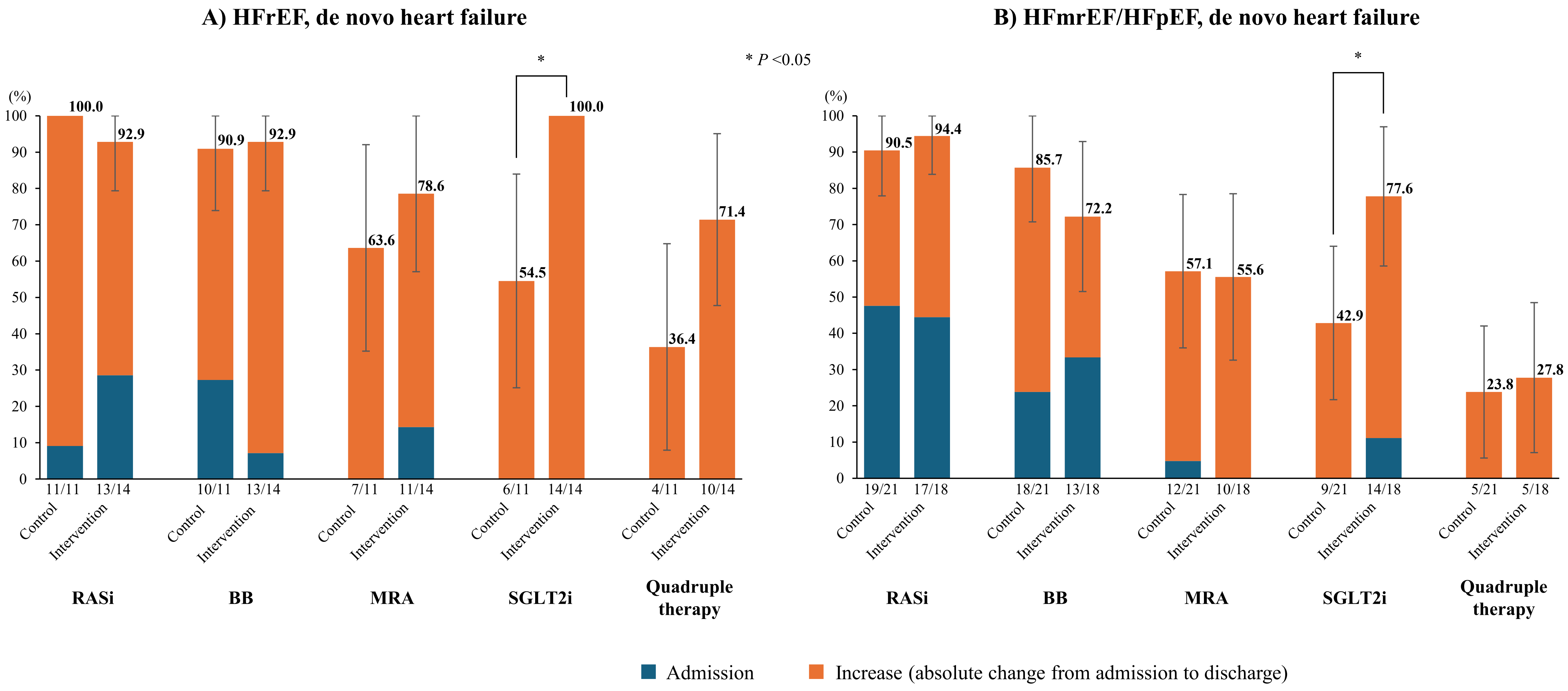

Orange segments represent the absolute increase in prescription rates from admission to discharge (discharge % − admission %)
Values are percentages. Error bars indicate 95% CIs for prescription rates at discharge; CIs are not shown for proportions of 100%.
Numbers below bars indicate n/N patients prescribed each agent (or quadruple therapy) at discharge.
