# Supplementary material for: Intensive pharmacist-led guideline-directed medical therapy for hospitalized heart failure patients: a single-center retrospective cohort study
Source: J Pharm Health Care Sci. 2026 Aug 3;12:93. doi: 10.1186/s40780-026-00616-9 (PMC13726016; doi:10.1186/s40780-026-00616-9)
Supplement: Supplementary file 3 [file 40780_2026_616_MOESM3_ESM.docx]

**Supplementary Tables**

Supplementary Table S1. Process indicators of checklist implementation in the intervention group

| Indicator | HFrEF (n = 23) | HFmrEF/HFpEF (n = 31) | Total (n = 54) |
| --- | --- | --- | --- |
| Usage metrics |  |  |  |
| Eligible hospitalizations, n | 23 | 31 | 54 |
| Checklists created, n (%) | 23 (100.0) | 30 (96.8) | 53 (98.1) |
| Checklists not created, n (%) | 0 (0.0) | 1 (3.2) | 1 (1.9) |
| Checklists initiated at intended timing, n (%) ^a^ | 23 (100.0) | 30 (100.0) | 53 (100.0) |
|  |  |  |  |
| Pharmacist recommendations |  |  |  |
| Patients with ≥1 direct GDMT recommendation, n (%) | 1 (4.3) | 2 (6.5) | 3 (5.6) |
| Accepted recommendations, n (%) ^b^ | 1 (100.0) | 1 (50.0) | 2 (66.7) |

^a^ Intended timing was defined as the time when all predefined initiation criteria described in the Methods were fulfilled (admission to the cardiology department for acute heart failure, “heart failure” as the primary diagnosis at admission, and enrollment in a heart failure rehabilitation program).

^b^ Percentages for “Accepted recommendations” were calculated using the number of direct GDMT recommendations as the denominator.
GDMT, guideline-directed medical therapy; HFmrEF, heart failure with mildly reduced ejection fraction; HFpEF, heart failure with preserved ejection fraction; HFrEF, heart failure with reduced ejection fraction

Supplementary Table S2a. Reasons for non-initiation of GDMT among patients with HFrEF

| Reason for non-initiation | RASi  Control  (n = 2) | RASi  Pharmacist-Led  Intervention  (n = 1) | BB  Control  (n = 3) | BB  Pharmacist-Led  Intervention  (n = 1) | MRA  Control  (n = 9) | MRA  Pharmacist-Led  Intervention  (n = 3) | SGLT2i  Control  (n = 6) | SGLT2i  Pharmacist-Led  Intervention  (n = 1) |
| --- | --- | --- | --- | --- | --- | --- | --- | --- |
| Hypotension | 1 (50.0%) | 0 | 0 | 0 | 0 | 0 | 0 | 0 |
| Hyperkalemia | 1 (50.0%) | 0 | 0 | 0 | 2 (22.2%) | 1 (33.3%) | 0 | 0 |
| Bradycardia | 0 | 0 | 0 | 1 (100.0%) | 0 | 0 | 0 | 0 |
| Unknown (not documented) | 0 | 1 (100.0%) | 3 (100.0%) | 0 | 7 (77.8%) | 2 (66.7%) | 6 (100.0%) | 1 (100.0%) |

Percentages are calculated using the number of patients who did not receive each GDMT class as the denominator.

Unknown/not documented indicates cases in which no specific reason for non-initiation was documented in the medical records.

ACEi, angiotensin‐converting enzyme inhibitor; ARB, angiotensin receptor blocker; ARNI, angiotensin receptor neprilysin inhibitor; BB, beta‐blocker; GDMT, guideline‐directed medical therapy; HFrEF, heart failure with reduced ejection fraction; RASi, renin–angiotensin system inhibitor (ACEi/ARB/ARNI); SGLT2i, sodium–glucose cotransporter-2 inhibitor

Supplementary Table S2b. Reasons for non-initiation of SGLT2i among patients with HFmrEF/HFpEF

| Reason for non-initiation | SGLT2i  Control (n = 15) | SGLT2i  Pharmacist-Led Intervention (n = 9) |
| --- | --- | --- |
| UTI | 0 | 1 (11.1%) |
| Unknown (not documented) | 15 (100.0%) | 8 (88.9%) |

Percentages are calculated using the number of patients who did not receive SGLT2i as the denominator.

Unknown/not documented indicates cases in which no specific reason for non-initiation was documented in the medical records.

HFmrEF, heart failure with mildly reduced ejection fraction; HFpEF, heart failure with preserved ejection fraction; SGLT2i, sodium–glucose cotransporter-2 inhibitor; UTI, urinary tract infection

Supplementary Table S3. Exploratory multivariable logistic regression analysis with robust standard errors for quadruple therapy use at discharge in patients with HFrEF

|  | Univariate analysis | | | Multivariate analysis | | |
| --- | --- | --- | --- | --- | --- | --- |
| Characteristic | Odds ratio | 95% CI | *P* value | Odds ratio | 95% CI | *P* value |
| Age (years) | 0.986 | 0.942–1.030 | 0.557 |  |  |  |
| Female | 0.357 | 0.097–1.310 | 0.120 |  |  |  |
| BMI (kg/m^2^) | 1.090 | 0.937–1.270 | 0.267 |  |  |  |
| Na (mmol/L) | 0.884 | 0.743–1.050 | 0.166 |  |  |  |
| K (mmol/L) | 1.130 | 0.436–2.930 | 0.801 |  |  |  |
| eGFR (mL/min/1.73 m^2^) | 0.985 | 0.952–1.020 | 0.368 |  |  |  |
| BNP (pg/mL) | 1.000 | 0.999–1.000 | 0.516 |  |  |  |
| sBP (mmHg) | 0.982 | 0.963–1.000 | 0.065 |  |  |  |
| dBP (mmHg) | 0.976 | 0.948–1.000 | 0.092 |  |  |  |
| HR (b.p.m.) | 0.971 | 0.944 | 0.040 |  |  |  |
| LVEF (%) | 0.998 | 0.915–1.090 | 0.972 | 0.967 | 0.876–1.070 | 0.506 |
| Hypertension | 0.571 | 0.145–2.250 | 0.423 |  |  |  |
| Dyslipidemia | 1.470 | 0.369–5.880 | 0.583 |  |  |  |
| Diabetes mellitus | 0.743 | 0.188–2.930 | 0.671 |  |  |  |
| De novo heart failure | 0.685 | 0.204–2.300 | 0.541 |  |  |  |
| Prior hospitalization for heart failure in 1 year | 1.140 | 0.235–5.490 | 0.874 |  |  |  |
| Ischemic heart disease | 6.400 | 1.220–33.500 | 0.028 | 4.300 | 0.853–21.700 | 0.077 |
| AF/AFL | 1.180 | 0.336–4.120 | 0.799 |  |  |  |
| Hospital stay (days) | 1.000 | 0.943–1.070 | 0.882 |  |  |  |
| Pharmacist-led intervention (vs control) | 5.200 | 1.410–19.200 | 0.013 | 5.130 | 1.100–24.000 | 0.038 |

Univariate odds ratios were obtained from logistic regression models with conventional standard errors. The multivariable model used robust standard errors.
In the multivariable model, only the intervention group indicator, history of ischemic heart disease, and LVEF were included as covariates; other variables were evaluated in univariate models only. Given the limited sample size, this analysis should be interpreted as exploratory.

AF, atrial fibrillation; AFL, atrial flutter; BMI, body mass index; BNP, brain natriuretic peptide; CI, confidence interval; dBP, diastolic blood pressure; eGFR, estimated glomerular filtration rate; HFrEF, heart failure with reduced ejection fraction; HR, heart rate; K, potassium; LVEF, left ventricular ejection fraction; Na, sodium; OR, odds ratio; sBP, systolic blood pressure

Supplementary Table S4. Stratified analyses of quadruple therapy use at discharge according to age group, sex, and renal function in patients with HFrEF

| Subgroup | Category | Control, n/N (%) | Pharmacist-Led Intervention, n/N (%) | Unadjusted OR (95% CI) | *P* value |
| --- | --- | --- | --- | --- | --- |
| Age group | ≥75 years | 4/14 (28.6) | 14/16 (87.5) | 17.50 (2.67–115.00) | 0.003 |
|  | <75 years | 5/8 (62.5) | 4/7 (57.1) | 0.80 (0.10–6.35) | 0.833 |
| Sex | Female | 1/7 (14.3) | 5/7 (71.4) | 15.00 (1.03–218.00) | 0.048 |
|  | Male | 8/15 (53.3) | 13/16 (81.2) | 3.79 (0.76–19.00) | 0.106 |
| Renal function (eGFR) | ≥45 mL/min/1.73 m² | 3/11 (27.3) | 10/12 (83.3) | 13.30 (1.78–100.00) | 0.012 |
|  | <45 mL/min/1.73 m² | 6/11 (54.5) | 8/11 (72.7) | 2.22 (0.38–13.20) | 0.379 |

Age was stratified as < 75 vs ≥ 75 years; sex as female vs male; and renal function as eGFR ≥ 45 vs < 45 mL/min/1.73 m².

Unadjusted odds ratios were obtained from logistic regression models fitted separately within each subgroup.

Given the limited sample size, these stratified analyses were considered exploratory and *P* values were not adjusted for multiple comparisons.

CI, confidence interval; eGFR, estimated glomerular filtration rate; OR, odds ratio; HFrEF, heart failure with reduced ejection fraction

Supplementary Table S5. Prescription of heart failure medications at admission and discharge in the control and pharmacist-led intervention groups among patients with de novo heart failure

|  | HFrEF (n = 25) | | | HFmrEF/HFpEF (n = 39) | | |
| --- | --- | --- | --- | --- | --- | --- |
| Medication | Control  (n = 11) | Pharmacist-Led Intervention (n = 14) | *P* value | Control  (n = 21) | Pharmacist-Led Intervention (n = 18) | *P* value |
| At admission |  |  |  |  |  |  |
| RASi (%) | 1 (9.1) | 4 (28.6) | 0.341 | 10 (47.6) | 8 (44.4) | 1 |
| BB (%) | 3 (27.3) | 1 (7.1) | 0.288 | 5 (23.8) | 6 (33.3) | 0.723 |
| MRA (%) | 0 (0) | 2 (14.3) | 0.487 | 1 (4.8) | 0 (0) | 1 |
| SGLT2i (%) | 0 (0) | 0 (0) | – | 0 (0) | 2 (11.1) | 0.206 |
| Quadruple therapy | 0 (0) | 0 (0) | – | 0 (0) | 0 (0) | – |
| At discharge |  |  |  |  |  |  |
| RASi (%) | 11 (100.0) | 13 (92.9) | 1 | 19 (90.5) | 17 (94.4) | 1 |
| BB (%) | 10 (90.9) | 13 (92.9) | 1 | 18 (85.7) | 13 (72.2) | 0.432 |
| MRA (%) | 7 (63.6) | 11 (78.6) | 0.656 | 12 (57.1) | 10 (55.6) | 1 |
| SGLT2i (%) | 6 (54.5) | 14 (100) | 0.009 | 9 (42.9) | 14 (77.6) | 0.049 |
| Quadruple therapy | 4 (36.4) | 10 (71.4) | 0.116 | 5 (23.8) | 5 (27.8) | 1 |

Data are presented as the number of cases (%).

*P* values were calculated using Fisher’s exact test.

–, *P* value not calculated because both groups had zero events.

De novo heart failure was defined as an index hospitalization for heart failure in patients without any prior history of heart failure.

Note: “Quadruple therapy” applies to HFrEF; for HFmrEF/HFpEF, the four-class combination (RASi, BB, MRA, and SGLT2i) is shown descriptively.

ACEi, angiotensin‐converting enzyme inhibitor; ARB, angiotensin receptor blocker; ARNI, angiotensin receptor neprilysin inhibitor; BB, beta‐blocker; HFmrEF, heart failure with mildly reduced ejection fraction; HFpEF, heart failure with preserved ejection fraction; HFrEF, heart failure with reduced ejection fraction; MRA, mineralocorticoid receptor antagonist; RASi, renin–angiotensin system inhibitor (ACEi/ARB/ARNI); SGLT2i, sodium–glucose cotransporter-2 inhibitor

Supplementary Table S6. Prescription of heart failure medications at admission and discharge in the control and pharmacist-led intervention groups among patients with prior heart failure

|  | HFrEF (n = 20) | | | HFmrEF/HFpEF (n = 19) | | |
| --- | --- | --- | --- | --- | --- | --- |
| Medication | Control  (n = 11) | Pharmacist-Led Intervention (n = 9) | *P* value | Control  (n = 6) | Pharmacist-Led Intervention (n = 13) | *P* value |
| At admission |  |  |  |  |  |  |
| RASi (%) | 8 (72.7) | 7 (77.8) | 1 | 5 (83.3) | 9 (69.2) | 1 |
| BB (%) | 7 (63.6) | 8 (88.9) | 0.319 | 5 (83.3) | 8 (61.5) | 0.605 |
| MRA (%) | 4 (36.4) | 6 (66.7) | 0.370 | 2 (33.3) | 3 (23.1) | 1 |
| SGLT2i (%) | 4 (36.4) | 6 (66.7) | 0.370 | 1 (16.7) | 4 (30.8) | 1 |
| Quadruple therapy | 2 (18.2) | 4 (44.4) | 0.336 | 0 (0) | 1 (7.7) | 1 |
| At discharge |  |  |  |  |  |  |
| RASi (%) | 9 (81.8) | 9 (100) | 1 | 5 (83.3) | 11 (84.6) | 1 |
| BB (%) | 9 (81.8) | 9 (100) | 1 | 5 (83.3) | 9 (69.2) | 1 |
| MRA (%) | 6 (54.5) | 9 (100) | 0.656 | 4 (66.7) | 7 (53.8) | 1 |
| SGLT2i (%) | 10 (90.9) | 8 (88.9) | 1 | 3 (50) | 8 (61.5) | 1 |
| Quadruple therapy | 5 (45.5) | 8 (88.9) | 0.116 | 2 (33.3) | 5 (38.5) | 1 |

Data are presented as the number of cases (%).

*P* values were calculated using Fisher’s exact test.

Prior heart failure was defined as hospitalization for worsening heart failure in patients with a documented history of heart failure before the index admission, regardless of previous hospitalizations.

Note: “Quadruple therapy” applies to HFrEF; for HFmrEF/HFpEF, the four-class combination (RASi, BB, MRA, and SGLT2i) is shown descriptively.

ACEi, angiotensin‐converting enzyme inhibitor; ARB, angiotensin receptor blocker; ARNI, angiotensin receptor neprilysin inhibitor; BB, beta‐blocker; HFmrEF, heart failure with mildly reduced ejection fraction; HFpEF, heart failure with preserved ejection fraction; HFrEF, heart failure with reduced ejection fraction; MRA, mineralocorticoid receptor antagonist; RASi, renin–angiotensin system inhibitor (ACEi/ARB/ARNI); SGLT2i, sodium–glucose cotransporter-2 inhibitor

Supplementary Table S7. Baseline characteristics of patients according to availability of 90-day follow-up data

|  | HFrEF | | | HFmrEF/HFpEF | | |
| --- | --- | --- | --- | --- | --- | --- |
| Characteristic | 90-day follow-up available (n = 31) | 90-day follow-up not available (n = 14) | *P* value | 90-day follow-up available (n = 28) | 90-day follow-up not available (n = 30) | *P* value |
| Age (years) | 76 (38–92) | 80 (37–89) | 0.297^a^ | 79 (51–94) | 83.5 (54–100) | 0.154^c^ |
| Female (%) | 10 (32.3) | 4 (28.6) | 1^b^ | 13 (46.4) | 16 (53.3) | 0.793^b^ |
| BMI (kg/m^2^) | 20.7 (15.3–42.5) | 21.0 (13.1–25.5) | 0.686^a^ | 23.9 (15.3–31.2) | 21.9 (14.9–34.1) | 0.417^c^ |
| Na (mmol/L) | 140 (132–145) | 140 (131–144) | 0.348^c^ | 140 (126–145) | 139 (126–145) | 0.563^a^ |
| K (mmol/L) | 4.1 (3.0–5.5) | 3.7 (2.7–4.3) | 0.009^c^ | 4.1 (3.0–5.6) | 4.1 (2.6–6.2) | 0.725^c^ |
| eGFR (mL/min/1.73 m^2^) | 46 (19–95) | 35 (24–76) | 0.249^a^ | 52 (23–90) | 45 (20–90) | 0.486^c^ |
| BNP (pg/mL) | 944.0 (111.2–4250.0) | 2188.1 (97.3–4250.0) | 0.066^a^ | 591.8 (45.3–2264.9) | 517.0 (133.5–2515.1) | 0.957^a^ |
| sBP (mmHg) | 129 (87–210) | 149 (113–206) | 0.111^c^ | 156 (95–220) | 147 (100–254) | 0.821^a^ |
| dBP (mmHg) | 81 (49–180) | 89 (59–135) | 0.098^a^ | 86 (50–139) | 84.5 (58–138) | 0.732^c^ |
| HR (b.p.m.) | 98 (50–154) | 103 (67–123) | 0.776^c^ | 98 (60–144) | 103 (42–185) | 0.282^c^ |
| LVEF (%) | 30 (12–39) | 34.5 (25–39) | 0.031^c^ | 48.5 (40.0–70.0) | 51.5 (40.0–79.0) | 0.159^a^ |
| Hypertension (%) | 20 (64.5) | 12 (85.7) | 0.176^b^ | 24 (85.7) | 22 (73.3) | 0.336^b^ |
| Dyslipidemia (%) | 8 (25.8) | 4 (28.6) | 1^b^ | 11 (39.3) | 12 (40.0) | 1^b^ |
| Diabetes mellitus (%) | 7 (22.6) | 4 (28.6) | 0.717^b^ | 12 (42.9) | 11 (36.7) | 0.789^b^ |
| Prior hospitalization for heart failure in 1 year (%) | 4 (12.9) | 4 (28.6) | 0.231^b^ | 2 (7.1) | 1 (3.3) | 0.605^b^ |
| Ischemic heart disease (%) | 12 (38.7) | 2 (14.3) | 0.165^b^ | 6 (21.4) | 5 (16.7) | 0.744^b^ |
| AF/AFL (%) | 11 (35.5) | 5 (35.7) | 1^b^ | 12 (42.9 | 17 (56.7) | 0.431^b^ |
| Hospital stay (days) | 23 (12–42) | 32 (19–57) | 0.078^c^ | 20 (8–49) | 21 (9–50) | 0.265^a^ |
| Medication (at admission) |  |  |  |  |  |  |
| RASi (%) | 14 (45.2) | 6 (42.9) | 1^b^ | 14 (50.0) | 18 (60.0) | 0.598^b^ |
| BB (%) | 14 (45.2) | 5 (35.7) | 0.746^b^ | 12 (42.9) | 12 (40.0) | 1^b^ |
| MRA (%) | 9 (29.0) | 3 (21.4) | 0.725^b^ | 2 (7.1) | 4 (13.3) | 0.671^b^ |
| SGLT2i (%) | 7 (22.6) | 3 (21.4) | 1^b^ | 4 (14.3) | 3 (10.0) | 0.701^b^ |
| Medication (at discharge) |  |  |  |  |  |  |
| RASi (%) | 29 (93.5) | 13 (92.9) | 1^b^ | 26 (92.9) | 26 (86.7) | 0.671^b^ |
| BB (%) | 29 (93.5) | 12 (85.7) | 0.578^b^ | 24 (85.7) | 21 (70.0) | 0.212^b^ |
| MRA (%) | 22 (71.0) | 11 (78.6) | 0.725^b^ | 15 (53.6) | 18 (60.0) | 0.791^b^ |
| SGLT2i (%) | 27 (87.1) | 11 (78.6) | 0.659^b^ | 18 (64.3) | 16 (53.3) | 0.435^b^ |
| Quadruple therapy | 20 (64.5) | 7 (50.0) | 0.512^b^ | 8 (28.6) | 9 (30.0) | 1^b^ |

Data are presented as the number of cases (%) or median (min–max).

^a^ Mann–Whitney *U* test; ^b^ Fisher’s exact test; ^c^ Student's *t*-test

Note: “Quadruple therapy” applies to HFrEF; for HFmrEF/HFpEF, the four-class combination (RASi, BB, MRA, and SGLT2i) is shown descriptively.

ACEi, angiotensin‐converting enzyme inhibitor; AF, atrial fibrillation; AFL, atrial flutter; ARB, angiotensin receptor blocker; ARNI, angiotensin receptor neprilysin inhibitor; BB, beta‐blocker; BMI, body mass index; BNP, brain natriuretic peptide; dBP, diastolic blood pressure; eGFR, estimated glomerular filtration rate; HFmrEF, heart failure with mildly reduced ejection fraction; HFpEF, heart failure with preserved ejection fraction; HFrEF, heart failure with reduced ejection fraction; HR, heart rate; K, potassium; LVEF, left ventricular ejection fraction; MRA, mineralocorticoid receptor antagonist; Na, sodium; RASi, renin–angiotensin system inhibitor (ACEi/ARB/ARNI); sBP, systolic blood pressure; SGLT2i, sodium–glucose cotransporter-2 inhibitor
