# Supplementary material for: Intensive pharmacist-led guideline-directed medical therapy for hospitalized heart failure patients: a single-center retrospective cohort study
Source: J Pharm Health Care Sci. 2026 Aug 3;12:93. doi: 10.1186/s40780-026-00616-9 (PMC13726016; doi:10.1186/s40780-026-00616-9)
Supplement: Supplementary file 4 [file 40780_2026_616_MOESM4_ESM.docx]

**Supplementary Fig. S1**. Prescription rates of heart failure medications at admission and discharge in the control group and the pharmacist-led checklist intervention group among patients with de novo heart failure. Panel A shows patients with HFrEF, and panel B shows patients with HFmrEF/HFpEF. Bars are stacked to show prescription rates at admission (bottom) and the absolute increase during hospitalization (top), with the total height representing prescription rates at discharge. Error bars indicate 95% confidence intervals (CIs) for prescription rates at discharge; CIs are not shown for proportions of 100%. Numbers below bars indicate n/N patients prescribed each agent (or quadruple therapy) at discharge.
Note: “Quadruple therapy” applies to HFrEF; for HFmrEF/HFpEF, the four-class combination (RASi, BB, MRA, and SGLT2i) is shown descriptively.

ACEi, angiotensin-converting enzyme inhibitor; ARB, angiotensin receptor blocker; ARNI, angiotensin receptor neprilysin inhibitor; BB, beta-blocker; CI, confidence interval; HFmrEF, heart failure with mildly reduced ejection fraction; HFpEF, heart failure with preserved ejection fraction; HFrEF, heart failure with reduced ejection fraction; MRA, mineralocorticoid receptor antagonist; RASi, renin–angiotensin system inhibitor (ACEi/ARB/ARNI); SGLT2i, sodium–glucose cotransporter-2 inhibitor

**Supplementary Fig. S2**. Prescription rates of heart failure medications at admission and discharge in the control group and the pharmacist-led checklist intervention group among patients with prior heart failure. Panel A shows patients with HFrEF, and panel B shows patients with HFmrEF/HFpEF. Bars are stacked to show prescription rates at admission (bottom) and the absolute increase during hospitalization (top), with the total height representing prescription rates at discharge. Error bars indicate 95% confidence intervals (CIs) for prescription rates at discharge; CIs are not shown for proportions of 100%. Numbers below bars indicate n/N patients prescribed each agent (or quadruple therapy) at discharge.
Note: “Quadruple therapy” applies to HFrEF; for HFmrEF/HFpEF, the four-class combination (RASi, BB, MRA, and SGLT2i) is shown descriptively.

ACEi, angiotensin-converting enzyme inhibitor; ARB, angiotensin receptor blocker; ARNI, angiotensin receptor neprilysin inhibitor; BB, beta-blocker; CI, confidence interval; HFmrEF, heart failure with mildly reduced ejection fraction; HFpEF, heart failure with preserved ejection fraction; HFrEF, heart failure with reduced ejection fraction; MRA, mineralocorticoid receptor antagonist; RASi, renin–angiotensin system inhibitor (ACEi/ARB/ARNI); SGLT2i, sodium–glucose cotransporter-2 inhibitor
